# Prospective observational study to assess the feasibility and safety of appropriate Plasmodium vivax radical cure with tafenoquine or primaquine after quantitative G6PD testing during pilot implementation in Thailand

**DOI:** 10.1136/bmjgh-2024-016720

**Published:** 2025-04-24

**Authors:** Prayuth Sudathip, Nardlada Khantikul, Aungkana Saejeng, Stephan Duparc, Penny Grewal Daumerie, Caroline Lynch, Elodie Jambert, Saowanee Viboonsanti, Darin Areechokchai, Jerdsuda Kanjanasuwan, Thannika Thong-ard, Panupong Kowsurat, Isabelle Borghini-Fuhrer, Chantana Padungtod

**Affiliations:** 1 Department of Disease Control, Ministry of Public Health, Nonthaburi, Thailand; 2 The Office of Disease Prevention and Control 1 Chiangmai, Division of Vector Borne Diseases, Ministry of Public Health, Nonthaburi, Thailand; 3 MMV Medicines for Malaria Venture, Geneva, Switzerland; 4 ImpactPlus, Geneva, Switzerland; 5 Independent regional advisor, Chaing Mai, Thailand

**Keywords:** Malaria, Other diagnostic or tool, Parasitology, Public Health

## Abstract

**Introduction:**

*Plasmodium vivax* recurrence prevention using tafenoquine or primaquine is critical for achieving Thailand’s malaria elimination targets. Both drugs may cause haemolysis in glucose-6-phosphate dehydrogenase (G6PD) deficient individuals. This study evaluated the operational feasibility and safety of administering tafenoquine or primaquine after quantitative G6PD point-of-care testing in Thailand.

**Methods:**

This prospective, observational, multicentre, longitudinal study was conducted between 23 May 2022 and 14 September 2023 during pilot implementation at seven sites in Yala and Mae Hong Son provinces. Eligible patients were ≥16 years old with uncomplicated *P. vivax* malaria. G6PD enzyme activity was quantified using a point-of-care device. All patients received 3-day chloroquine plus (based on G6PD enzyme activity): single-dose tafenoquine 300 mg (≥6.1 U/g Hb), or primaquine 15 mg/day for 14 days (≥4.1 U/g Hb), or primaquine 45 mg/week for 8 weeks (≤4.0 U/g Hb), with follow-up on days 5 and 14. Hospital admissions were reviewed to confirm acute haemolytic anaemia cases. The primary endpoint was the percentage of *P. vivax* patients ≥16 years old treated or not treated with tafenoquine in accordance with G6PD enzyme activity.

**Results:**

Of 316 *P*. *vivax* patients screened, 187 were enrolled. All patients completed quantitative G6PD testing. According to G6PD status, appropriate use or non-use was 100% (95% CI 97.2, 100 (132/132)) with tafenoquine, 100% (95% CI 96.5, 100 (104/104)) with daily primaquine and 99.5% (97.1, 100 (186/187)) with weekly primaquine. At day 5, adverse events possibly related to haemolysis occurred in 46.3% (37/80) of patients with tafenoquine, 56.8% (46/81) with daily primaquine and 77.8% (14/18) with weekly primaquine, with no confirmed drug-induced acute haemolytic anaemia cases.

**Conclusion:**

Point-of-care quantitative G6PD testing prior to appropriate tafenoquine or primaquine administration was operationally feasible within the Thailand health system, with no concerning adverse events, supporting implementation of this treatment algorithm in areas of active *P. vivax* transmission.

**WHAT IS ALREADY KNOWN ON THIS TOPIC**

- To prevent the recurrence of *Plasmodium vivax* malaria requires the administration of an 8-aminoquinoline, either tafenoquine or primaquine.
- Quantitative G6PD testing is required to exclude patients at risk of acute haemolytic anaemia following treatment with tafenoquine or primaquine.

**WHAT THIS STUDY ADDS**

- This real-world study of a pilot implementation demonstrates that quantitative G6PD testing before administering tafenoquine or primaquine is operationally feasible within the Thailand public health service.

**HOW THIS STUDY MIGHT AFFECT RESEARCH, PRACTICE OR POLICY**

- This study provides evidence to support the wider implementation of quantitative G6PD testing, single-dose tafenoquine, and daily or weekly primaquine in six provinces in Thailand that are currently experiencing *P. vivax* malaria outbreaks.
- The study has important implications for the elimination of malaria in Thailand and the prevention of re-establishment of transmission in areas where *P. vivax* has been eliminated.

## Introduction

 Considerable progress has been made towards Thailand’s goal to eliminate all human malaria by 2026, with reported cases declining from 46 895 in 2012 to 3216 in 2021.^1 2^ However, the deteriorating security situation in Myanmar interrupted malaria services and displaced persons across the border into Thailand. Since 2021, malaria cases have increased in Thailand, particularly in the western Myanmar border region. In 2022, there were 10 161 reported malaria cases, of which 94.5% were *Plasmodium vivax* and more than a third were imported, mainly from Myanmar.^1^ In 2023, malaria cases continued to increase, despite real-time case-based surveillance and full implementation of the 1-3-7 strategy, that is, rapid case notification within 1 day, case investigation within 3 days and foci response within 7 days.^3 4^ To regain and consolidate progress towards malaria elimination and prevent re-establishment of transmission, new tools and approaches are required to address *P. vivax* malaria in Thailand.^5^

*P. vivax* is refractive to malaria control and elimination primarily because the parasite has a dormant liver stage, the hypnozoite. Hypnozoites are undetectable and asymptomatic until reactivating to cause acute clinical malaria episodes (relapses). In Thailand, the first relapse typically occurs within 3–4 weeks of the primary infection, but a single infectious bite can result in multiple relapses over months or even years.^6^ Repeated relapses erode patient health, elevate the malaria burden, expand health system costs and promote onward malaria transmission.^7^,^9^ Hypnozoites also allow the parasite to be silently carried within the human host, with the potential to cause outbreaks in areas where *P. vivax* has been previously eliminated. Drugs for the treatment of acute malaria are ineffective against hypnozoites. To fully cure a patient with *P. vivax*, termed radical cure, requires administration of a blood schizonticide to address the acute blood-stage infection plus an 8-aminoquinoline, primaquine or tafenoquine to address the hypnozoite stage and thereby prevent relapse.

The Thailand National Treatment Guidelines (2021) recommend first-line treatment of uncomplicated *P. vivax* malaria with 3-day chloroquine plus primaquine (0.25 mg/kg/day) for 14 days. Primaquine has high efficacy in Thailand when adherence to the 14-day regimen is maintained,^10^ though poor adherence undermines treatment effectiveness.^11^ Additionally, primaquine efficacy is diminished in individuals who have impaired P450 2D6 (CYP2D6) activity, and around half of malaria patients may have poor or intermediate CYP2D6 metaboliser phenotypes in Thailand.^12^

The single-dose aminoquinoline tafenoquine was granted conditional approval by the Thailand Food and Drug Administration in December 2019 but has not yet been deployed in the public health system. In randomised clinical trials, chloroquine plus tafenoquine (300 mg) reduced *P. vivax* malaria recurrence over 6 months by 70% versus chloroquine plus placebo and had similar efficacy to chloroquine plus primaquine 15 mg/day for 14 days.^13 14^ Tafenoquine requires coadministration with chloroquine, so cannot be used with an artemisinin-based schizonticide, such as dihydroartemisinin-piperaquine.^15^ Clinical evidence suggests that tafenoquine efficacy is not affected by CYP2D6 polymorphisms.^16^

Glucose-6-phosphate dehydrogenase (G6PD) deficiency is an inherited enzymopathy common in malaria-endemic regions, with an estimated prevalence of 13.6% in Thailand.^17^ Both primaquine and tafenoquine may cause haemolysis in G6PD-deficient individuals, which in severe cases can lead to acute haemolytic anaemia (AHA).^18^ To ensure patient safety, G6PD testing is required before primaquine or tafenoquine to guide appropriate therapy.

In 2017, Thailand and India were the first malaria-endemic countries to approve a point-of-care quantitative G6PD test. Since its inclusion in the Thailand Clinical Practice Guidelines for Malaria Case Management in 2019, the quantitative G6PD test has been routinely used at higher-level health facilities, where healthcare providers have been trained to support *P. vivax* radical cure with primaquine. Where quantitative G6PD testing is not available, *P. vivax* patients are referred to these facilities for radical cure.

The Assessing Radical Cure Treatment in Routine Care (ARCTIC) study assessed the operational feasibility and safety within the Thailand public health system of *P. vivax* radical cure incorporating quantitative G6PD testing to guide appropriate treatment with tafenoquine or primaquine. The study was conducted during pilot implementation in Yala and Mae Hong Son provinces to inform revisions to the national malaria treatment policy to safely deploy tafenoquine, with the aim of accelerating malaria elimination and containing *P. vivax* outbreaks. A short film outlining the study is available online.^19^

## Methods

### Study context

Malaria control and prevention in Thailand are managed by 39 provincial-level Division of Vector Borne Diseases (DVBD) centres and 301 district-level units, supported by 329 malaria clinics and approximately 1.04 million village health volunteers.^20^ Malaria services are delivered through the DVBD’s network of specialised malaria clinics as part of a vertical programme and via general healthcare facilities at district and provincial hospitals.^21^ Village health workers are involved in health education, case detection and follow-up. Thailand has an established real-time electronic case-based malaria reporting system to support elimination, with active follow-up of patients. As well as the 1-3-7 strategy for case management,^3 4^ integrated drug efficacy surveillance (iDES) has been implemented, with follow-up of *P. vivax* cases at day 14, 29, 60 and 90 post treatment to ensure cure. Thus, the infrastructure for identifying and intensively following patients is well established.^22^

Yala is the southernmost province in Thailand, bordering Malaysia, and a hotspot for malaria transmission, with dense forest supporting vector populations and a highly mobile human population.^23^ Mae Hong Son province in northern Thailand has historically had one of the highest malaria incidences in Thailand.^24^ Remote and densely forested, the province has recently experienced an influx of refugees from civil unrest in Myanmar who have limited access to malaria services. In 2022 and 2023, the annual *P. vivax* malaria incidence/1000 population was 0.15 and 0.30 in Yala and 5.91 and 6.85 in Mae Hong Son, respectively.^25^

### Study design

**Figure 1:**
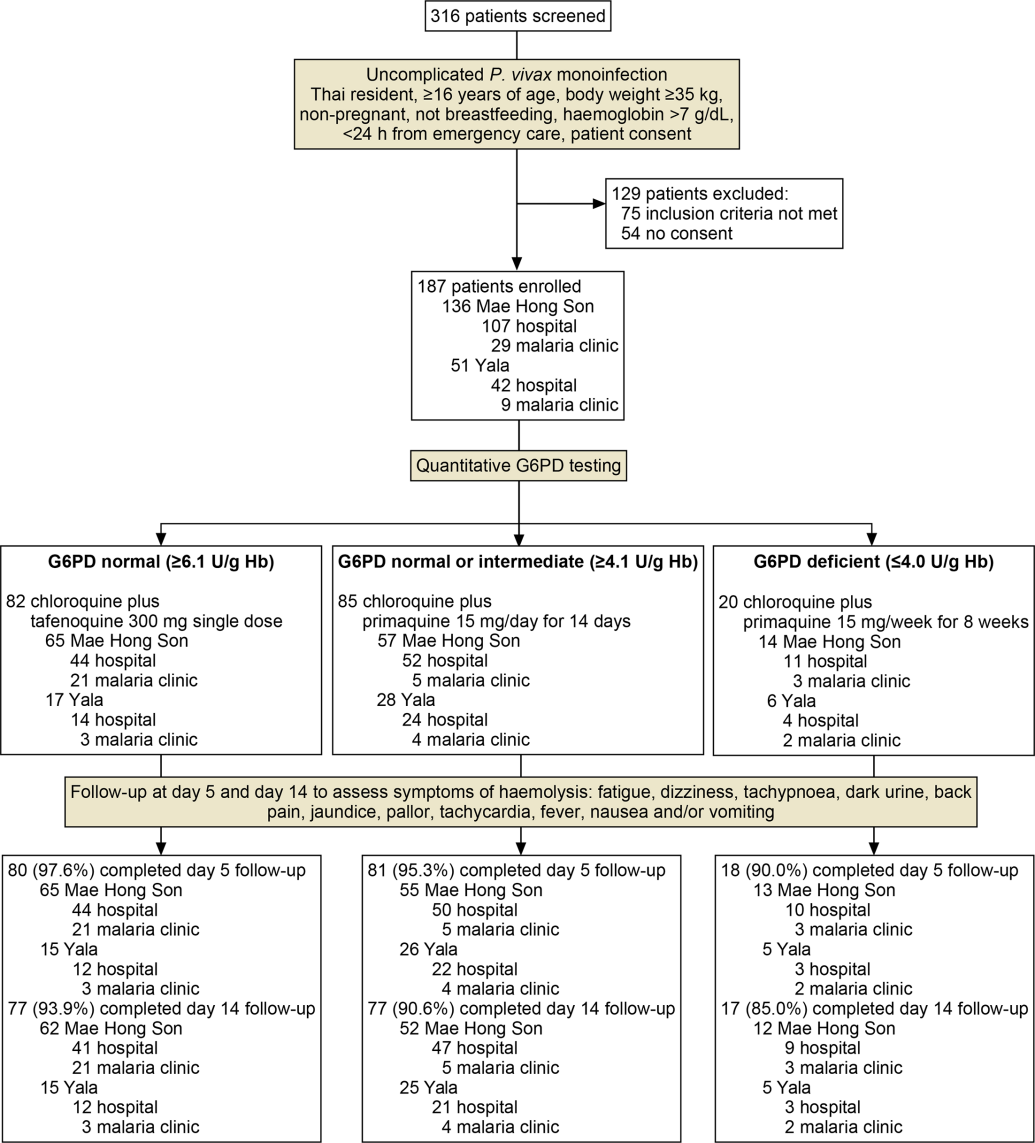
Study overview and patient disposition. Hospitals were community hospitals within the general health system serving one district, whereas malaria clinics are run by the Division of Vector Borne Diseases within the National Malaria Programme. In all cases, patients not completing follow-up on days 5 and 14 were because they did not return on the specified days. G6PD, glucose-6-phosphate dehydrogenase.

This prospective, observational, multicentre, longitudinal study assessed the operational feasibility and safety of a revised treatment algorithm for *P. vivax* radical cure incorporating tafenoquine or primaquine following quantitative G6PD testing (figure 1). The study was conducted in two phases. The first phase (23 May 2022 to 31 December 2022) was implemented at five community hospitals in Yala province (Than To, Bannang Sata, Kabang) and two community hospitals in Mae Hong Son province (Mae Sariang, Sop Moei). Community hospitals are the smallest hospitals, located at the district level, providing primary care treatment within individual districts. Following an interim analysis of 50 patients, the independent study oversight committee approved progression to the second phase, involving continued enrolment at the community hospitals included in phase one plus implementation in malaria clinics (lower-level facilities) within 3 hours of hospital access. Two malaria clinics recruited patients, Than To in Yala and Mae Sariang in Mae Hong Son, while two additional malaria clinics (Bannang Sata and Mueang in Yala) received training but were unable to recruit patients. The second phase was conducted between 1 January 2023 and 6 February 2023 and, following an ethical committee approval to increase the sample size, also between 1 June 2023 and 14 September 2023. The study is registered at ClinicalTrials.gov with the identifier NCT05753150. The protocol is available from the corresponding author on request.

### Quantitative test and drugs

The STANDARD G6PD test (SD Biosensor, Suwon-si, Republic of Korea) quantifies G6PD enzyme activity relative to haemoglobin level, using 10 µL of capillary or venous blood samples, providing results within 2 min on a portable analyser.^26^ Based on the manufacturers’ instructions, individuals were categorised based on their G6PD enzyme activity as G6PD normal (≥6.1 U/g Hb), G6PD intermediate (4.1–6.0 U/g Hb) or G6PD deficient (≤4.0 U/g Hb).

Study drugs were chloroquine (250 mg chloroquine phosphate tablets (150 mg base) or 500 mg chloroquine phosphate tablets (300 mg base); Premed Pharma Plus Company Limited, Bangkok, Thailand), tafenoquine (150 mg tablets; GSK, Ware, UK) and primaquine (primaquine phosphate 26.3 mg tablets (15 mg base); Government Pharmaceutical Organisation, Bangkok, Thailand or 13.2 mg tablets (7.5 mg base); Remedica, Limassol, Cyprus).

In the community hospital setting, quantitative G6PD tests were already available. Additional G6PD tests and tafenoquine were supplied to health facilities by the DVBD using routine supply routes for antimalarial diagnostics and drugs. Primaquine and other antimalarial drugs were already available in the health system.

### Participants

Eligible patients were Thailand permanent residents, ≥16 years of age, with uncomplicated malaria, bodyweight ≥35 kg, haemoglobin >7 g/dL and microscopically-confirmed *P. vivax* monoinfection, residing <24 hour travel from an emergency care facility (figure 1). Exclusion criteria were current participation in a clinical trial, severe or complicated malaria, pregnancy or lactation, diabetes treated with metformin or G6PD-deficient patients who were being treated with sulfonylurea drugs. All eligible consenting patients were prospectively enrolled. There was no randomisation or sampling.

### Patient and public involvement

Patients and the public were not involved in the study design or conduct and were recruited as part of the routine management of malaria following informed consent. The DVBD sponsored the study, was involved in all aspects of the study and led the pilot implementation.

### Intervention

Before each phase, the DVBD organised training for relevant staff at participating sites (for training overview see online supplemental appendix methods S1). Training included the quantitative G6PD test procedure, updated radical cure treatment algorithm, haemolysis symptoms, reporting procedures and patient counselling. Standard operating procedures were provided for identification of suspected AHA, initial patient management and transfer to a referral hospital (online supplemental appendix methods S2 and S3). Training was supported by comprehensive materials intended for national scale-up of tafenoquine implementation as well as study-specific materials (eg, see online supplemental appendix methods S3 and S4). Refresher training was conducted after tafenoquine was resupplied to the sites in January 2023 and again after the pause for the approval of the study extension. Supervision and monitoring were delivered by DVBD staff and site investigators who were part of the health system structure.

All patients provided informed consent and received counselling regarding *P. vivax* radical cure prior to G6PD testing and drug administration according to a checklist (online supplemental appendix methods S5), supported by educational materials (eg, online supplemental appendix methods S6). For eligible patients, on day 0, demographic and baseline clinical characteristics were recorded, the quantitative G6PD test conducted, and treatment initiated as per the *P. vivax* radical cure treatment algorithm (figure 1). Patients ≥50 kg bodyweight received chloroquine 600 mg/day (base) administered on days 0 and 1 and 300 mg/day on day 2, and those weighing 35–49 kg were administered 600 mg on day 0 and 150 mg on days 1 and 2. The 8-aminoquinolines were administered from day 0 according to G6PD enzyme activity: G6PD normal (≥6.1 U/g Hb) tafenoquine 300 mg single dose; G6PD normal or intermediate (≥4.1 U/g Hb), primaquine 15 mg/day for 14 days and G6PD deficient (≤4.0 U/g Hb) primaquine 45 mg/week for 8 weeks. For G6PD normal patients, tafenoquine was to be given preferentially rather than primaquine. All drugs were administered orally, and the first dose was to be observed. Patients were requested to return for follow-up on day 5 (±1 day), which was introduced for the pilot implementation, in addition to the routine visit on day 14 (−1/+2 days), with study completion on day 14. Consistent with routine care within the health system, patients received telephone call reminders for follow-up appointments and 300 Baht travel compensation for the day 14 visit, which was extended for the day 5 visit.

At the day 5 and day 14 visits, adverse events of special interest (AESI) possibly associated with haemolysis were assessed using a symptom checklist and a clinical decision framework developed for application to routine case management (online supplemental appendix methods S3). As per this framework, adverse event severity and criteria for suspected AHA were evaluated by a specialist physician. If the physician suspected AHA, the patient was transferred for further investigation to either the Yala Referral Hospital or the Mae Hong Son Provincial Hospital, which have the appropriate facilities to effectively manage AHA. As these two facilities were likely to see all hospitalisations for AHA in the study districts, study staff at the referral hospitals screened all admissions presenting with AHA signs, renal failure and/or requiring blood transfusion for patient records with national identifying numbers matching patients included in the study.

### Data management

The standard malaria clinical reporting form used by the health service was updated to align with the revised treatment algorithm. Patient data were captured on case report forms and entered into the study database by municipal staff. Automated checks were done to detect missing data or discrepancies, which were resolved by the investigators, and any amendments were tracked and approved—tasks performed by a DVBD supervisor. The database was locked before the final statistical analysis was done. All data were stored and evaluated per regulatory requirements and applicable guidance for electronic records.

### Endpoints

The primary endpoint was the percentage of *P. vivax* patients ≥16 years old treated or not treated with tafenoquine in accordance with the appropriate level of G6PD enzyme activity, calculated as:


$$\frac{\text{Appropriate use of tafenoquine}+\text{appropriate non-use of tafenoquine}}{\text{Total use of tafenoquine}+\text{appropriate non-use of tafenoquine}}$$

Calculated in a similar manner, a secondary endpoint was the percentage of *P. vivax* patients ≥16 years old treated or not treated with primaquine in accordance with the appropriate level of G6PD enzyme activity. Additional secondary endpoints were the percentage of *P. vivax* patients who received tafenoquine or primaquine based on the correct application of the treatment algorithm (ie, G6PD status and age) and the frequency of confirmed drug-induced AHA.

Safety data were collected prospectively, focusing on AESI, including those possibly associated with haemolysis (fatigue, dizziness, tachypnoea, dark urine, back pain, jaundice, pallor, tachycardia, fever, nausea and/or vomiting) and altered liver function following antimalarial therapy (alanine aminotransferase (ALT) ≥3×the upper limit of normal (ULN) and total bilirubin ≥2×ULN or ALT ≥3×ULN and International Normalised Ratio >1.5). Adverse event severity and relationship to drug treatment were assessed. Serious adverse events and treatment and hospitalisation for AHA were also reported.

A posthoc analysis investigated all patients with symptoms and signs of haemolysis reported on the day 5 or 14 follow-up visits to determine if they met the criteria for suspected AHA, defined as new onset dark-coloured or red urine or new onset jaundice or pallor plus at least one of the following signs: fatigue, dizziness, shortness of breath or back pain or a serious adverse event of hospitalisation, blood transfusion, renal replacement therapy or death.

### Statistical analysis

The study size calculation was based on the primary endpoint of the study. Assuming the enrolment of 110 patients, the 95% CIs for a percentage of 99% of patients treated or not treated appropriately with tafenoquine would be 97.1 to 100, with a precision of 1.9%. There was no limit on the number of patients enrolled at each health centre.

The analysis population included all enrolled patients, and the safety population included all patients who were treated with tafenoquine or primaquine, with no imputation for missing values. Tafenoquine was out of stock from 1 September 2022 to 31 December 2022 because of an international supply issue, and patients recruited during this period could only receive primaquine, so they were omitted from the primary analysis.

Planned subgroup analyses were according to antimalarial treatment, facility level, age class, sex, G6PD status, weight category and municipality. Outcomes were reported using descriptive statistics, with two-sided 95% CIs (Clopper-Pearson). Baseline was defined as the day of treatment initiation (day 1) with follow-up visit dates calculated accordingly (day 5, day 14). As this was a real-world study, patients who returned outside the follow-up windows (until day 12 for the day 5 visit instead of day 5±1 day) were included. Statistical analysis was performed using SAS software V.9.4 (SAS Institute, Cary, North Carolina, USA).

## Results

### Participants

Of 316 *P*. *vivax* patients presenting, 187 were enrolled, 51 (27.3%) from Yala and 136 (72.7%) from Mae Hong Son (figure 1). Patient characteristics are shown in table 1. 79.7% (149/187) of patients were treated at community hospitals and all were ≥16 years of age. Of the 49 females enrolled, 29 (59.2%) were of childbearing potential; none were breastfeeding or pregnant. All patients were included in the analysis and safety populations; 50 patients enrolled and treated with primaquine while tafenoquine was unavailable were excluded from the primary analysis of tafenoquine’s appropriate use and non-use.

**Table 1:** Demographic and baseline clinical characteristics

Characteristic	Tafenoquine (n=82)	Primaquine, 7 days (n=85)	Primaquine, 8 weeks (n=20)	All (n=187)
Community hospitals	58 (70.7)	76 (89.4)	15 (75.0)	149 (79.7)
Malaria clinics	24 (29.3)	9 (10.6)	5 (25.0)	38 (20.3)
Mean age (SD) (range), years	39.0 (14.6) (17–86)	38.8 (17.2) (16–97)	37.2 (12.1) (17–60)	38.7 (15.6) (16–97)
Sex				
Male	60 (73.2)	67 (78.8)	11 (55.0)	138 (73.8)
Female	22 (26.8)	18 (21.2)	9 (45.0)	49 (26.2)
Female of childbearing potential	13 (59.0)	9 (50.0)	7 (77.8)	29 (59.2)
Pregnant	0	0	0	0
Breastfeeding	0	0	0	0
Age class, years				
≥16 to ≤24	15 (18.3)	19 (22.4)	2 (10.0)	36 (19.3)
25 to ≤44	41 (50.0)	38 (44.7)	12 (60.0)	91 (48.7)
45 to ≤64	22 (26.8)	21 (24.7)	6 (30.0)	49 (26.2)
≥65	4 (4.9)	7 (8.2)	0	11 (5.9)
Weight in class				
<50	10 (12.2)	13 (15.3)	5 (25.0)	28 (15.0)
≥50 to <70	61 (74.4)	50 (58.8)	11 (55.0)	122 (65.2)
≥70 to <90	10 (12.2)	20 (23.5)	4 (20.0)	34 (18.2)
≥90 to <120	1 (1.2)	2 (2.4)	0	3 (1.6)
≥120	0	0	0	0
Occupation				
Child/student	1 (1.2)	5 (5.9)	2 (10.0)	8 (4.3)
Soldier/police	2 (2.4)	10 (11.8)	1 (5.0)	13 (7.0)
Government employee	3 (3.7)	3 (3.5)	1 (5.0)	7 (3.7)
Vendor	0	1 (1.2)	1 (5.0)	2 (1.1)
Fruit farmer	2 (2.4)	6 (7.1)	1 (5.0)	9 (4.8)
Other types of farming	41 (50.0)	27 (31.8)	8 (40.0)	76 (40.6)
Rubber tapper	13 (15.9)	6 (7.1)	0	19 (10.2)
Rice farmer	2 (2.4)	3 (3.5)	0	5 (2.7)
Other	17 (20.7)	16 (18.8)	4 (20.0)	37 (19.8)
Forest clearing	0	1 (1.2)	0	1 (0.5)
Fisherman	0	0	0	0
Freelance	1 (1.2)	4 (4.7)	0	5 (2.7)
None	0	3 (3.5)	2 (10.0)	5 (2.7)
Mean time since symptoms appeared (SD) (range), days	3.7 (2.66) (0–14)	3.9 (2.32) (0–11)	4.5 (2.93) (0–10)	3.9 (2.54) (0–14)
Diagnosis by microscopy	82 (100)	85 (100)	20 (100)	187 (100)
Mean haemoglobin (SD) (range), g/dL	13.1 (1.79) (8.1, 17.8)	13.3 (1.90) (7.2, 17.4)	12.6 (2.52) (8.6, 19.4)	13.2 (1.93) (7.2, 19.4)
Malaria in the previous 3 months	1 (1.2)	4 (4.7)	0	5 (2.7)
*P. vivax*	1 (1.2)	4 (4.7)	0	5 (2.7)
*P. falciparum*	0	0	0	0

Data are n (%) unless otherwise stated.

Most patients completed day 5 follow-up (95.7%; 179/187) and day 14 follow-up (91.4%; 171/187). The first treatment dose was directly observed for 80.1% (149/186) of those prescribed chloroquine, 97.6% (80/82) for tafenoquine, 77.6% (66/85) for daily primaquine and 65.0% (13/20) for weekly primaquine. One patient who received daily primaquine was treated with dihydroartemisinin-piperaquine rather than chloroquine.

### G6PD quantitative testing

Quantitative G6PD test results were available for all 187 enrolled patients. All 82 patients who received tafenoquine were G6PD normal (online supplemental appendix figure S1A). Of the 85 patients who received daily primaquine, 31.8% (27/85) were G6PD normal and 68.2% (58/85) were G6PD intermediate. Of the 20 patients who received weekly primaquine, 5.0% (1/20) were G6PD intermediate and 95.0% (19/20) were G6PD deficient (online supplemental appendix figure S1A). The G6PD intermediate patient who received weekly primaquine was enrolled at a community hospital. Median (IQR) G6PD activity was 7.3 (6.6, 8.0) U/g Hb for patients who received tafenoquine, 5.8 (5.3, 6.3) U/g Hb for daily primaquine and 2.4 (2.0, 3.4) U/g Hb for weekly primaquine. G6PD deficiency prevalence was 10.2% (19/187) and was higher in females (16.3%; 8/49) than in males (8.0%; 11/138) (online supplemental appendix figures S1B, S1C). Of the males, 31.9% (44/138) were G6PD intermediate compared with 30.6% (15/49) of females (online supplemental appendix figure S1B).

### Primary outcome

For the primary outcome, according to G6PD status, appropriate use or non-use of tafenoquine was 100% (95% CI 97.2, 100 (132/132)) (table 2). The results were consistent across community hospitals and malaria clinics (table 2). No children <16 years of age, or pregnant or breastfeeding women were enrolled, so the treatment algorithm was applied correctly for all patients (100%; 95% CI 95.6, 100 (82/82)) receiving tafenoquine.

**Table 2:** Tafenoquine appropriate use and appropriate non-use according to G6PD activity by facility level and overall

Outcome	Community hospitals (n=149)	Malaria clinics (n=38)	Overall (n=187)
Number of patients treated when tafenoquine available*	99 (66.4)	38 (100.0)	137 (73.3)
Total TQ use, n (%)	58 (58.6)	24 (63.2)	82 (59.9)
TQ appropriate use	58 (58.6)	24 (63.2)	82 (59.9)
TQ inappropriate use	0	0	0
TQ appropriate non-use, n (%)†	36 (36.4)	14 (36.8)	50 (36.5)
TQ appropriate use and appropriate non-use‡, n/N	94/94	38/38	132/132
TQ appropriate use and appropriate non-use‡, % (95% CI)	100 (96.2, 100)	100 (90.8, 100)	100 (97.2, 100)

* Tafenoquine was out of stock from 1st1 September 2022 to 31st December 2022, and 50 patients patients enrolled during this period could only be treated with primaquine and were therefore excluded from the primary analysis.

† Patients with G6PD enzyme levels <6.1 U/g Hb.

‡ Calculated as: (tafenoquine appropriate use+tafenoquine appropriate non-use) / (Ttotal tafenoquine use+tafenoquine appropriate non-use).

G6PD, glucose-6-phosphate dehydrogenase; TQ, tafenoquine.

### Secondary outcomes

According to G6PD status, appropriate use or non-use of daily primaquine was 100% (95% CI 96.5, 100 (104/104)) (table 3). No children <16 years of age or pregnant or breastfeeding women were enrolled, so the treatment algorithm was applied correctly for all patients (100%; 95% CI 95.8, 100 (85/85)) receiving daily primaquine.

**Table 3:** Daily and weekly primaquine appropriate use and appropriate non-use according to G6PD activity by facility level and overall

Outcome	Community hospitals (n=149)	Malaria clinics (n=38)	Overall (n=187)
Daily PQ			
Total daily PQ use, n (%)	76 (51.0)	9 (23.7)	85 (45.5)
Daily PQ appropriate use	76 (51.0)	9 (23.7)	85 (45.5)
Daily PQ inappropriate use	0	0	0
Daily PQ appropriate non-use, n (%)*	14 (9.4)	5 (13.2)	19 (10.2)
Daily PQ appropriate use and appropriate non-use†, n/N	90/90	14/14	104/104
Daily PQ appropriate use and appropriate non-use†, % (95% CI)	100 (96.0, 100)	100 (76.8, 100)	100 (96.5, 100)
Weekly PQ			
Total weekly PQ use, n (%)	15 (10.1)	5 (13.2)	20 (10.7)
Weekly PQ appropriate use	14 (9.3)	5 (13.2)	19 (10.1)
Weekly PQ inappropriate use	1 (0.7)	0	1 (0.5)
Weekly PQ appropriate non-use, n (%)‡	134 (89.9)	33 (86.8)	167 (89.3)
Weekly PQ appropriate use and appropriate non-use†, n/N	148/149	38/38	186/187
Weekly PQ appropriate use and appropriate non-use†, % (95% CI)	99.3 (96.3, 100)	100 (90.8, 100)	99.5 (97.1, 100)

* Patients with G6PD enzyme levels <4.1 U/g Hb.

† Calculated as: (primaquine appropriate use+primaquine appropriate non-use) / (Ttotal primaquine use+primaquine appropriate non-use).

‡ Patients with G6PD enzyme levels >4.0 U/g Hb.

PQ, primaquine.

For weekly primaquine, appropriate use or non-use according to G6PD status was 99.5% (97.1, 99.9 (186/187)) (table 3). One patient who should have received daily primaquine received weekly primaquine. No children <16 years of age or pregnant or breastfeeding women were enrolled, so the treatment algorithm was applied correctly to 95.0% (95% CI 75.1, 99.9 (19/20)) of patients receiving weekly primaquine.

### Safety

Day 5 follow-up was completed by 95.7% (179/187) of patients—94.6% (141/149) from community hospitals and 100% (38/38) from malaria clinics. Day 14 follow-up was completed by 91.4% (171/187) of patients (figure 1). Microscopy was performed for 89.8% (168/187) of patients on day 14 and all were negative for *P. vivax* and other species of malaria, including *P. knowlesi*.

#### Adverse events of special interest

**Figure 2:**
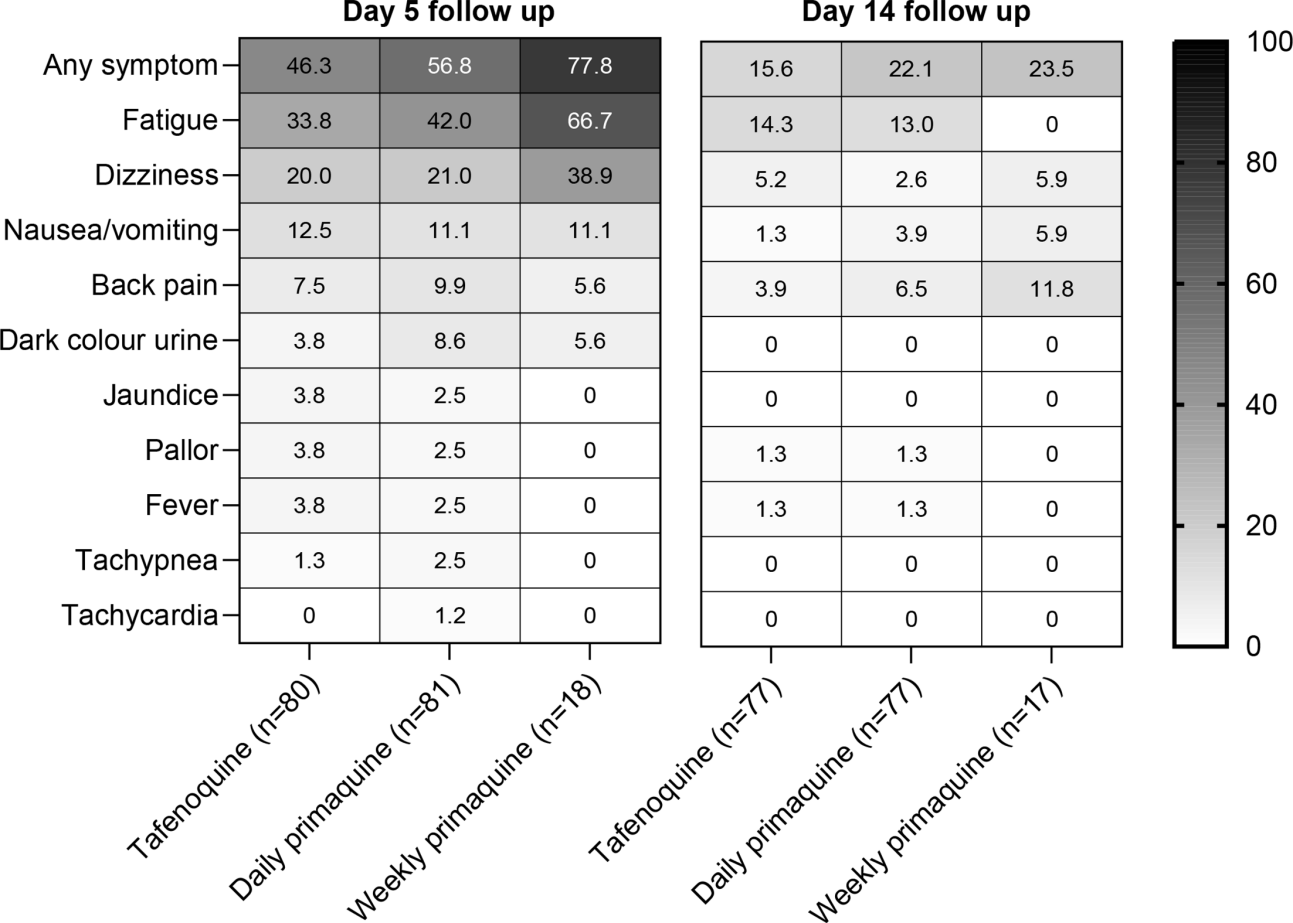
Frequency of adverse events of special interest potentially associated with haemolysis. Data are the percentage of patients by treatment group at the day 5 and day 14 follow-up visits.

There were no reports of liver dysfunction in any treatment group on days 5 or 14. On day 5, AESIs possibly associated with haemolysis were reported for 54.2% (97/179) of patients overall: 46.3% (37/80) with tafenoquine, 56.8% (46/81) with daily primaquine and 77.8% (14/18) with weekly primaquine (figure 2). The most frequent AESI was fatigue, both overall (40.8%; 73/179) and within each treatment group (figure 2). On day 14, AESIs possibly associated with haemolysis were reported by 19.3% (33/171) of patients, all of which were of mild severity: 15.6% (12/77) with tafenoquine, 22.1% (17/77) with daily primaquine and 23.5% (4/17) with weekly primaquine (figure 2). Fatigue was the most common AESI on day 14, occurring in 12.3% (21/171) of patients, and was also the most common AESI in the tafenoquine (13.0%; 10/77) and daily primaquine groups (14.3%; 11/77), but back pain was the most common AESI in the weekly primaquine group (11.8%; 2/17) (figure 2).

A posthoc analysis of the 97 patients with AESI possibly associated with haemolysis identified 14 cases of suspected AHA on day 5 (dark urine, pallor or yellowing of the skin/sclera). Six had been treated with tafenoquine (7.5%; 6/80), seven with daily primaquine (8.6%; 7/81) and one with weekly primaquine (5.6%; 1/18) (online supplemental appendix table S1). Clinical review of these cases did not confirm drug-induced AHA, with all patients receiving appropriate therapy based on their G6PD enzyme activity. All of these patients attended both day 5 and day 14 visits, and none of them met the criteria for suspected AHA on day 14.

#### Other adverse events

Twenty-four other adverse events were reported during the study, mainly headache (62.5%; 15/24) (online supplemental appendix table S2). Most adverse events reported on day 5 were mild (96.1%; 98/102). In the tafenoquine group, three patients had moderate adverse events (3.8%; 3/80), and one had a severe adverse event related to a scrub typhus infection (1.3%; 1/80), which was also deemed a serious adverse event (see below).

The frequency of adverse events on day 5 was similar between community hospitals (57.4%; 81/141) and malaria clinics (55.3%; 21/38), but their nature differed. For example, dizziness was reported for 42.1% (16/38) of patients from malaria clinics versus 17.0% (24/141) from community hospitals (online supplemental appendix figure S2). A similar proportion of men (55.2%; 74/134) and women (62.2%; 28/45) reported adverse events on day 5, with no obvious differences between the nature of the adverse events (online supplemental appendix figure S3).

#### Serious adverse events

Four serious adverse events were reported, none of which were considered treatment related by the prescribing physician, and all four patients made a full recovery. Full narratives are included in the supplementary appendix (online supplemental appendix S1 narratives). Briefly, in the tafenoquine group, three serious adverse events occurred in G6PD normal patients, including one case of thalassaemia trait with blood transfusion, one case of leptospirosis and one case of scrub typhus. In the daily primaquine group, one serious adverse event occurred in a G6PD intermediate patient with severe malaria and severe thrombocytopenia requiring transfusion with leucocyte-depleted pooled platelet concentrate for idiopathic thrombocytopenic purpura. Screening of patient records for the referral hospitals did not identify any additional patients admitted following treatment with tafenoquine or primaquine.

## Discussion

In this pilot implementation of *P. vivax* radical cure at community hospitals and malaria clinics in Yala and Mae Hong Son provinces, all 187 *P*. *vivax* patients enrolled underwent G6PD testing and 100% received appropriate treatment or non-treatment with tafenoquine or daily primaquine, and 99.5% with weekly primaquine. There were no confirmed drug-induced AHA cases, treatment-related serious adverse events or notable differences in the adverse event profile for tafenoquine or daily primaquine.

G6PD deficiency prevalence was 10.2% (19/187) and higher in females than in males, an observation described previously in southern Thailand.^27^ In Thailand, the most common G6PD variants are Mahidol^487G>A^ and Viangchan^871G>A^,^28^,^34^ with median G6PD activities in hemizygous males of 10.2% and 7.5% that of normal males, respectively.^35^ The high G6PD deficiency prevalence and low G6PD enzyme activities of the most common G6PD variants have impeded *P. vivax* radical cure implementation.^36 37^ However, the reasonable caution that healthcare providers have towards prescribing 8-aminoquinolines may also explain the high adherence to G6PD testing and appropriate prescribing in this study.

Five patients who received daily primaquine would have been eligible for tafenoquine according to the treatment algorithm. Four were treated in the first 3 months of implementation, possibly indicating a delay in tafenoquine adoption by some healthcare providers. Real-time data available to the DVBD allows continued monitoring of adherence to G6PD testing and 8-aminoquinolines prescribing to identify issues and deploy responses, such as additional training and supervision or retraining. Also, the current study may be a useful tool in providing reassurance to clinicians of the haemolytic safety of tafenoquine in G6PD normal *P. vivax* patients.

Given the known haematological responses to primaquine and tafenoquine, clinically relevant haemolysis should be identifiable at day 5 following treatment initiation.^18 38^ However, as *P. vivax* malaria causes anaemia, symptoms such as fatigue, dizziness, nausea, etc are likely to be commonly experienced by patients following an acute malaria episode.^7^ Posthoc review of all cases with AHA symptoms found 14 cases with suspected AHA, all of which had been treated appropriately based on G6PD enzyme activities. For operational implementation, ongoing AHA monitoring will be incorporated into routine pharmacovigilance activities including passive AHA case detection plus assessments at two routine follow-up visits on day 7 and day 14.

Patient adherence to follow-up attendance was high; only 4.3% of patients (8/187) did not attend on day 5 and 8.6% (16/187) on day 14. Patient counselling emphasised the need to assess haemolysis symptoms on days 5 and 14 and cure on day 14, with telephone reminders, and patients were compensated for travel costs, in accordance with current policy. These follow-up rates are high compared with other settings. In Brazil, for example, during the implementation of *P. vivax* radical cure, 25.9% (837/3228) of patients returned on day 5.^39^ However, in Brazil, there was no systematic follow-up of vivax patients before implementing the new treatment algorithm. In Thailand, patients are used to being contacted and returning to the clinic following a diagnosis of malaria, with intensive follow-up incorporated in the 1-3-7 strategy and iDES. Additionally, patient education and follow-up are supported by community health workers, awareness of G6PD deficiency is well developed in Thailand, and the case load is relatively low. The patient population was Thai residents living within 24 hours of an emergency care facility, and follow-up rates would be expected to be lower in migrant and more remote populations.

Other countries in the Greater Mekong subregion are in the process of implementing quantitative G6PD testing. In Cambodia, village health workers trained in point-of-care quantitative G6PD testing generated results which closely correlated with those obtained by laboratory technicians.^40 41^ Similar to the current study, a phased approach was adopted, with real-time data collection and careful monitoring of adverse events with clear referral pathways in case of complications.^42^ This built confidence in the safety of the procedures among health workers, and along with sensitisation of the community, the *P. vivax* radical cure protocol was well accepted by staff and patients and is now being scaled up across Cambodia.^42^ In Vietnam, similar to the current study, there was high adherence to quantitative G6PD testing following training.^43^ Regular quality control and supervision following training supported skills retention, but refresher training every 3 months was suggested for areas with low case numbers.

To our knowledge, the only other operational study concerning the implementation of quantitative G6PD testing with tafenoquine was conducted in Brazil. Similar to Thailand, there was 100% adherence to G6PD testing before tafenoquine administration; 99.7% (95% CI 99.4, 99.8; 4664/4680) of *P. vivax* patients correctly received tafenoquine according to G6PD status, with no cases of treatment-related AHA.^39^ Note that in Brazil, the operational effectiveness of tafenoquine and primaquine was at least as high as clinical efficacy observed in phase 3 randomised clinical trials.^44^ This implementation was also phased, with higher, then lower healthcare facilities trained.^45^ After the initial training, some healthcare providers lacked confidence in conducting quantitative G6PD testing and prescribing the tafenoquine regimen, prompting adjustments to improve the understanding of G6PD testing and the treatment algorithm.^45^ Additionally, knowledge gaps were addressed through onsite training, peer communication via a messaging app, and the use of educational materials.^45^

This observational study is subject to potential limitations such as selection bias and confounding due to the absence of randomisation. Although conducted in a real-world context, to meet ethical review board requirements, it was necessary to obtain written consent for data to be used, which may have influenced patient or provider behaviour. The patients may not be representative of all individuals with *P. vivax* malaria in the Yala and Mae Hong Son provinces. Additionally, there is a limitation related to attrition bias, with 8.6% (16/187) of enrolled patients not completing follow-up. In this study, there was no provision to assess the effectiveness of tafenoquine or primaquine for the prevention of *P. vivax* relapse, though clinical efficacy was shown in two phase 3 studies including sites in Thailand; with tafenoquine, recurrence-free efficacy at 6 months was 73.3% (95% CI, 64.7 to 80.2) and 62.4% (54.9 to 69.0); and with primaquine 15 mg/day for 14 days, it was 76.0% (95% CI, 64.6 to 84.1) and 69.6% (95% CI, 60.2 to 77.1).^13 14^ For comparison, recurrence-free efficacy with chloroquine plus placebo was 27.7% (95% CI, 19.6 to 36.6).^14^ The effectiveness of the new *P*. *vivax* radical cure treatment algorithm will be routinely monitored through iDES.

The situation along the Thailand–Myanmar border, with a substantial influx of refugees and limited access to healthcare, threatens to undermine the progress that Thailand and Myanmar have made towards malaria elimination. In the six Thai provinces that border Myanmar (Tak, Mae Hong Son, Kanchanaburi, Ratchaburi, Prachup Kirikhan and Petchaburi), outbreak response strategies have been implemented to strengthen case detection and improve follow-up rates. The roles of village health volunteers are being expanded to include proactive case detection and behaviour change communication activities for malaria prevention. Targeted chemoprevention and *P. vivax* radical cure with near-patient G6PD testing and single-dose tafenoquine or primaquine are essential interventions to contain the *P. vivax* outbreak in this highly mobile and vulnerable patient population. Thailand is also currently assessing the implementation of 7-day primaquine (0.5 mg/kg), with pilot studies in higher-level health centres. Mass drug administration of chloroquine is also being piloted in high burden areas.

The use of quantitative G6PD testing prior to tafenoquine or primaquine was operationally feasible and had acceptable safety when deployed in community hospitals and malaria clinics in Thailand. Consequently, the Thailand Ministry of Public Health has taken the decision to fully implement this radical cure treatment algorithm across the six provinces along the Thailand–Myanmar border. Implementation of *P. vivax* radical cure is a key element in reversing the intensity and spread of the current outbreak, accelerating malaria elimination, and preventing the re-establishment of transmission where *P. vivax* has already been eliminated.

## Supplementary material

10.1136/bmjgh-2024-016720 online supplemental file 1

## Data Availability

Data are available upon reasonable request.
