# Supplementary material for: Prospective observational study to assess the feasibility and safety of appropriate Plasmodium vivax radical cure with tafenoquine or primaquine after quantitative G6PD testing during pilot implementation in Thailand
Source: BMJ Glob Health. 2025 Apr 24;10(4):e016720. doi: 10.1136/bmjgh-2024-016720 (PMC12035454; doi:10.1136/bmjgh-2024-016720)
Supplement: online supplemental file 1 [file bmjgh-10-4-s001.pdf]

Supplementary appendix

Prospective observational study to assess the feasibility and safety of appropriate *Plasmodium vivax* radical cure with tafenoquine or primaquine after quantitative G6PD testing during pilot implementation in Thailand

Prayuth Sudathip; Nardlada Khantikul; Aungkana Saejeng; Stephan Duparc; Penny Grewal Daumerie; Caroline Lynch; Elodie Jambert; Saowanee Viboonsanti; Darin Areechokchai; Jerdsuda Kanchanasuwan, Thannika Thong-ard; Panupong Kowsurat; Isabelle Borghini-Fuhrer; Chantana Padungtod

Methods S1. Training overview 2

Methods S2. Guidance for identification and management of AHA-suspected cases 3

Methods S3: Supportive poster summarising guidance on recognising the symptoms of acute haemolytic anaemia (AHA) and the required response 5

Methods S4. Examples of training support materials 7

Methods S5. Checklist for patient counselling 8

Methods S6. Example of patient educational materials 9

Figure S1. Quantitative G6PD testing results 10

Table S1. Patients with suspected acute haemolytic anaemia at the day 5 follow-up visit (post-hoc analysis) 11

Table S2. Other adverse events reported at day 5 and day 14 follow-up visits 12

Figure S2. Adverse events of any nature reported by patients during day 5 and day 14 follow-up visits by facility level 13

Figure S3. Adverse events of any nature reported by patients during day 5 and day 14 follow-up visits by sex 14

S1 Narratives. Safety narratives for patients with serious adverse events 15

Methods S1. Training overview

<table>
<tr>
<td>Training delivery

Department for Vector Borne Diseases

Training participants
Yala
• 44 Hospital staff
  ○ 12 doctors
  ○ 15 nurses
  ○ 17 [Medical technologist, Pharmacists and Epidemiologists]
• 12 Malaria clinic staff
  ○ 7 Community Health Workers
  ○ 3 Microscopist
  ○ 2 Public health officers
Mae Hong Son
• 29 Hospital staff
  ○ 8 doctors
  ○ 8 nurses
  ○ 13 [Medical technologist, Pharmacists and Epidemiologists]
• 6 Malaria clinic staff
  ○ 3 Community Health Workers
  ○ 1 Microscopist
  ○ 2 Public health officers</td>
<td>Training content

P. vivax lifecycle and the importance of radical cure

G6PD testing procedures
• 21 hours of practical training
• 13 hours of supervision

Treatment algorithm
• Patient eligibility
• Tafenoquine and primaquine regimens
• G6PD-based prescribing

Safety
• Symptoms of haemolysis
• Assessment and referral procedures

Reporting
• Updates to routine reporting forms

Patient counselling
• Study purpose and consent procedure*
• Purpose of G6PD test
• Treatment options
• Recognising the symptoms of haemolysis and appropriate actions
• Importance of adherence to primaquine
• Importance of day 5 and 14 follow-up visits</td>
</tr>
<tr>
<td colspan="2">Training timeline
• Phase 1: Community hospitals only (23 May 2022)
• Phase 2: Community hospitals plus malaria clinics (recommended by Independent Study Oversight Committee, December 2022)
• End of recruitment (31 August 2023)</td>
</tr>
</table>

*Study-specific training. All other training was designed to be used across the Thailand public health system.

Methods S2. Guidance for identification and management of AHA-suspected cases

<u>**Identification of project participants at risk of Acute Haemolytic Anaemia (AHA)**</u>
During a scheduled follow-up visit or a visit outside of schedule by a project participant presenting him/herself to a health facility, check for symptoms/signs listed in the adverse events checklist below:

**Adverse Events (AE) Checklist**	
**Symptoms & Signs that may trigger Adverse Events of Special Interest (AESI)**	**Response**
**1. Fatigue**	Keep patient under observation. Check for increase in severity and/or adverse events combining. Hospitalise if fear of developing AHA.
**2. Dizziness**	
**3. Breathlessness or shortness of breath (tachypnea)**	
**4. Dark (red or black) urine**	
**5. Back pain**	
**6. Yellowing of the skin and/or sclera (jaundice)**	
**7. Pallor**	
**8. Rapid heart rate (tachycardia)**	
**9. Fever**	
**10. Nausea and/or vomiting**	

<u>**Initial management of suspected AHA cases**</u>
In case AHA risk is suspected, and if the patient is not at a hospital, arrange to transfer him/her to the nearest hospital (which may be a district hospital) for further assessment and initial clinical support based on general AHA management guidance per the checklist below. This includes possible referral for blood transfusion at a higher-level HF.

**Checklist for Clinical Management of AHA***
1. Oral hydration
2. Keep in in-patient facility
3. Request clinical assessment by a physician on duty
4. Check blood group, haemoglobin (Hb) and/or haematocrit
5. Check plasma/serum creatinine or Blood Urea Nitrogen (BUN), if possible
6. Consider blood transfusion* following local/hospital guidelines or as per WHO guidelines**
*Blood transfusion will be performed at Yala Referral Hospital or Mae Hong Son Provincial Hospital under this project.<br>**WHO 2016<br>- Hb <7 g/dl: transfuse (with packed red cells)<br>- Hb <9 g/dl with concurrent haemolysis: transfuse (with packed red cells)<br>- Hb 7-9 g/dl or >9 g/dl, and no evidence of concurrent haemolysis: provide careful fluid management and monitoring of urine colour and Hb level.

<u>**Administrative Guidance for Transfer of Patient to Yala Referral Hospital or to Mae Hong Son Provincial Hospital** (and elsewhere)</u>
Following clinical assessment, the physician may decide to:

- Allow the patient to go home, educate him/her to observe AHA signs/symptoms and report to project staff.
- Admit the patient to the district hospital for observation.

- Consider patient transfer to Yala Referral Hospital or Mae Hong Son Provincial Hospital urgently by following the following guidance:

3.1 Notify project hospital co-investigator: Director of the district hospital
3.2 Notify project field coordinators: [contact details provided].
3.3 If the decision of the physician-in-charge at the district hospital and that of the hospital director is to refer the patient to Yala Hospital then please contact [contact details for specialist haematologist provided] or Sri Sangwan Hospital then please contact [contact details for specialist nephrologist provided].
3.4 Arrange for patient transfer by hospital ambulance for further clinical evaluation and critical care management including blood transfusion, if necessary. Travel time for the ambulance to these referral hospitals would be approximately 3 hours.

Methods S3: Supportive poster summarising guidance on recognising the symptoms of acute haemolytic anaemia (AHA) and the required response

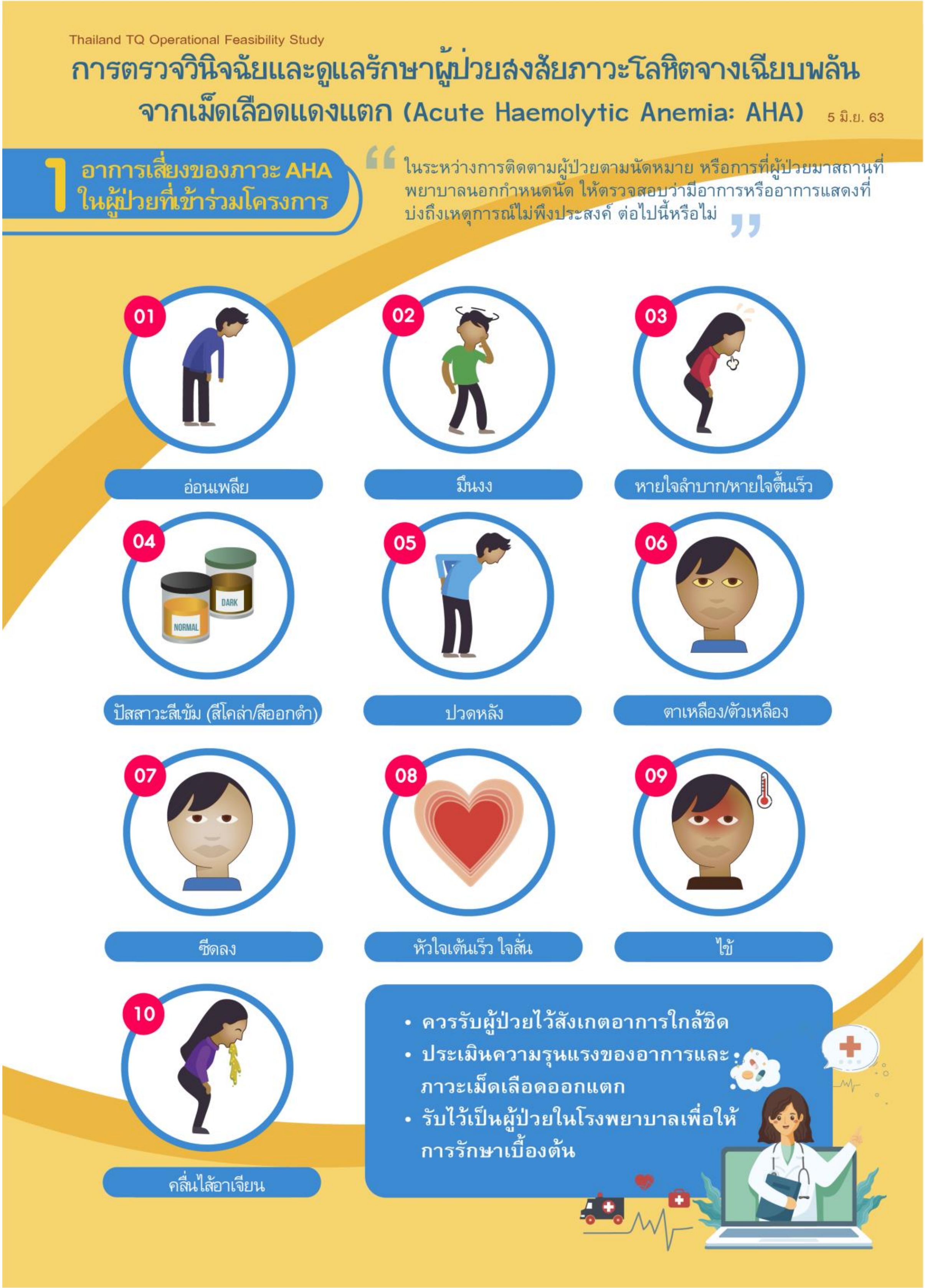

การตรวจวินิจฉัยและดูแลรักษาผู้ป่วยสงสัยภาวะโลหิตจางเฉียบพลันจากเม็ดเลือดแดงแตก (Acute Haemolytic Anemia: AHA)

5 มิ.ย. 63

2 การดูแลรักษาเบื้องต้นสำหรับผู้ป่วยสงสัยภาวะ AHA

“ กรณีสงสัยภาวะ AHA ให้จัดการส่งต่อผู้ป่วยไปยังโรงพยาบาลที่ใกล้ที่สุด เพื่อให้แพทย์ประเมินอาการและให้การรักษาเบื้องต้นตามแนวทางข้างล่างนี้ รวมถึงอาจปรึกษาแพทย์ผู้เชี่ยวชาญที่ รพศ. เพื่อส่งต่อผู้ป่วยไปรับการถ่ายเลือดหากมีอาการรุนแรง ”

ข้อปฏิบัติทางการแพทย์ ในการรักษาผู้ป่วย AHA*	
1.	ให้สารน้ำทางปาก
2.	รับไว้เป็นผู้ป่วยในโรงพยาบาล
3.	ให้แพทย์เวรประเมินอาการทางคลินิก
4.	ตรวจหาหมู่เลือด ระดับฮีโมโกลบิน และฮีมาโตคริต
5.	ตรวจระดับการทำงานของไต BUN, Cr
6.	พิจารณาให้เลือด *ตามแนวทางขององค์การอนามัยโลก 2016**

*หมายเหตุ สำหรับผู้ป่วยที่เข้าร่วมโครงการนี้ การให้เลือดจะทำที่โรงพยาบาลศูนย์ยะลาเท่านั้น

**แนวทางการให้เลือดตาม WHO 2016
- Hb <7 g/dl: ให้เลือด packed red cells
- Hb <9 g/dl ที่มีภาวะเม็ดเลือดแดงแตกร่วมด้วย : ให้เลือด packed red cells
- Hb 7-9 g/dl or >9 g/dl, และไม่มีสัญญาณที่บ่งชี้ถึงภาวะเม็ดเลือดแดงแตก
: ให้สารน้ำอย่าระมัดระวังและคอยติดตามดูสีปัสสาวะและระดับฮีโมโกลบินอย่างใกล้ชิด

3 แนวทางการจัดการส่งต่อผู้ป่วยไปยัง รพศ.ยะลา

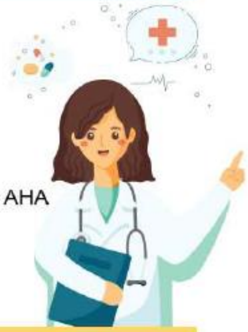

จากการประเมินอาการทางคลินิกของผู้ป่วย แพทย์อาจพิจารณาที่จะ:
- อนุญาตให้ผู้ป่วยกลับบ้านได้ สอนให้ผู้ป่วยสังเกตอาการ/อาการแสดงของ AHA และรายงานต่อเจ้าหน้าที่โครงการ
- รับผู้ป่วยไว้ ในโรงพยาบาลชุมชนเพื่อสังเกตอาการ
- พิจารณาส่งต่อผู้ป่ วยไป รพศ.ยะลา ตามคำแนะนำต่อไปนี้

3.1 แจ้งผู้อำนวยการโรงพยาบาล
นพ....................................... โทร....................................)

3. 2 แจ้งผู้ประสานงานโครงการภาคสนามนายมะหะมะนอ บาโงปะแด (โทร 080-702 8614), ซึ่งจะแจ้งต่อไปยัง:น.พ. เฉลิมพล โอสถพรมมา (หัวหน้าโครงการร่วม, โทร 099 321 4156) และ/หรือ พ.ญ. ชีวนันท์ เลิศพิริยสุวัฒน์ (หัวหน้าโครงการ, โทร 089 920 6195)

3. 3 หากแพทย์ผู้ดูแลผู้ป่วยและผู้อำนวยการโรงพยาบาลตัดสินใจจะส่งต่อผู้ป่วยไปยัง รพศ.ยะลา ให้ติดต่อ พ.ญ. ฮามีดะห์ มูหามัด ผู้เชี่ยวชาญด้านอายุรกรรม-โลหิตวิทยา รพศ.ยะลา (โทร 086 288 9794)เพื่อขออนุญาตส่งต่อผู้ป่วยไปโดยด่วน

3. 4 จัดการส่งต่อผู้ป่วยโดยรถพยาบาลฉุกเฉิน เพื่อสามารถประเมินอาการเป็นระยะและเพื่อรับบริบาลผู้ป่วยวิกฤต รวมถึงการให้เลือดถ้าจำเป็น ทั้งนี้ควรส่งต่อโดยใช้เวลาเดินทางไม่เกิน 3 ชั่วโมงถึงโรงพยาบาลศูนย์

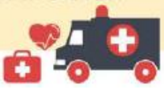

Methods S4. Examples of training support materials

Video clip G6PD testing<br><br>https://www.youtube.com/watch?v=w5Kzu4-XfFo	
G6PD testing guide	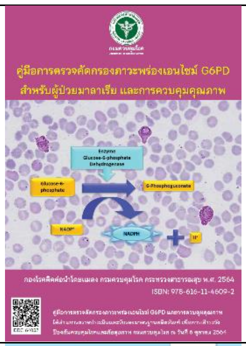
Operational manual of safety monitoring of antimalarial – Tafenoquine in Thailand for medical personnel.<br><br>คู่มือการดำเนินงานการเฝ้าระวังความปลอดภัยจากการใช้ยาต้านมาลาเรีย Tafenoquine ในประเทศไทยสำหรับบุคลากรทางการแพทย์	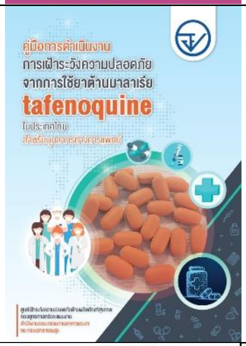
Job aid	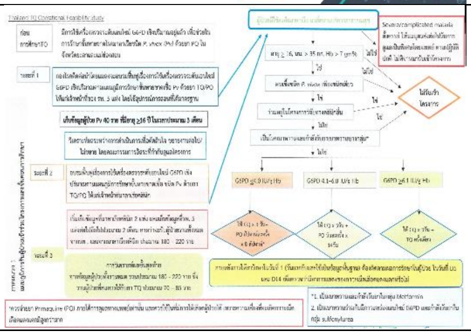
General slide decks	• เหตุการณ์ไม่พึงประสงค์ (Adverse Event: AEs)<br>• เหตุการณ์ไม่พึงประสงค์ร้ายแรง (Serious Adverse Event: SAEs)
Study specific slide decks	• Operational Feasibility of Appropriate *Plasmodium vivax* Radical Cure with Tafenoquine or Primaquine After Quantitative G6PD Testing in Thailand<br>• Operational guidelines of TQ project: workflow

Methods S5. Checklist for patient counselling

ภาคผนวก 5

บัญชีรายการให้คำปรึกษาผู้ป่วย

โครงการ: การศึกษาความเป็นไปได้ในทางปฏิบัติของการรักษาผู้ป่วยมาลาเรียชนิดไวแวกซ์ขั้นหายขาดอย่างเหมาะสมด้วยยาทาฟีโนควินหรือไพรมาควิน โดยใช้การตรวจระดับเอนไซม์ G6PD เชิงปริมาณ ประเทศไทย

___ อธิบายประโยชน์ของการจ่ายยา TQ และ PQ

___ สอบถามผู้ป่วยเรื่องประวัติการมีภาวะเม็ดเลือดแดงแตก หรือเลือดไหลไม่หยุด หรือเลือดออกมาก

___ แจ้งให้ผู้ป่วยทราบถึงความเสี่ยงของภาวะโลหิตจางชนิดเฉียบพลันจากเม็ดโลหิตแดงแตก (AHA) เมื่อได้รับยา TQ หรือ PQ

___ แนะนำให้ผู้ป่วยต้องตรวจดูสีปัสสาวะของตนเอง

___ แนะนำให้ผู้ป่วยหยุดยา PQ ทันทีหากมีปัสสาวะสีเข้มขึ้น

___ แจ้งให้ผู้ป่วยทราบว่าควรไปรับคำแนะนำทางแพทย์จากที่ใดหากเกิดมีปัสสาวะสีเข้มขึ้น

เอกสารอ้างอิง:

องค์การอนามัยโลก. การตรวจความพร่องเอนไซม์ G6PD เพื่อความปลอดภัยในการใช้ยาไพรมาควินสำหรับการรักษาขั้นหายขาดเชื้อมาลาเรียไวแวกซ์และโอวาเล่. เมืองเจนีวา: องค์การอนามัยโลก, คศ. 2016.

(WHO. Testing for G6PD deficiency for safe use of primaquine in radical cure of *P. vivax* and *P. ovale*. Geneva: World Health Organization, 2016.)

The Ethical Review Committee for Research in Human Subjects, Ministry of Public Health, Thailand

Date of Approved 11 MAY 2023

Methods S6. Example of patient educational materials

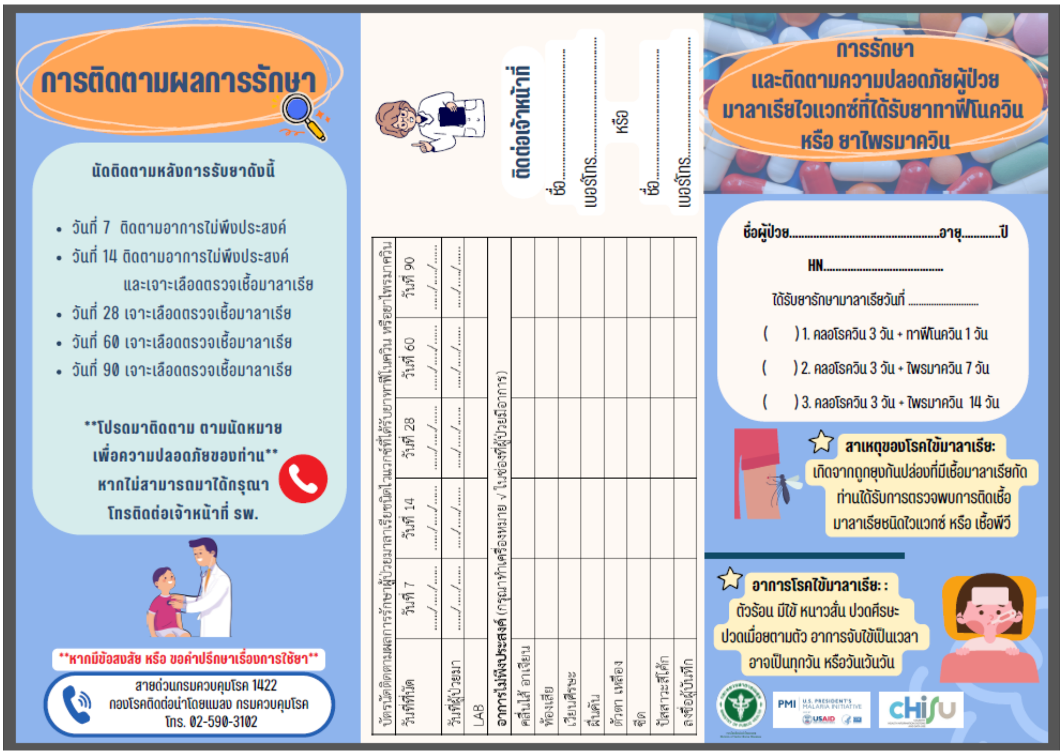

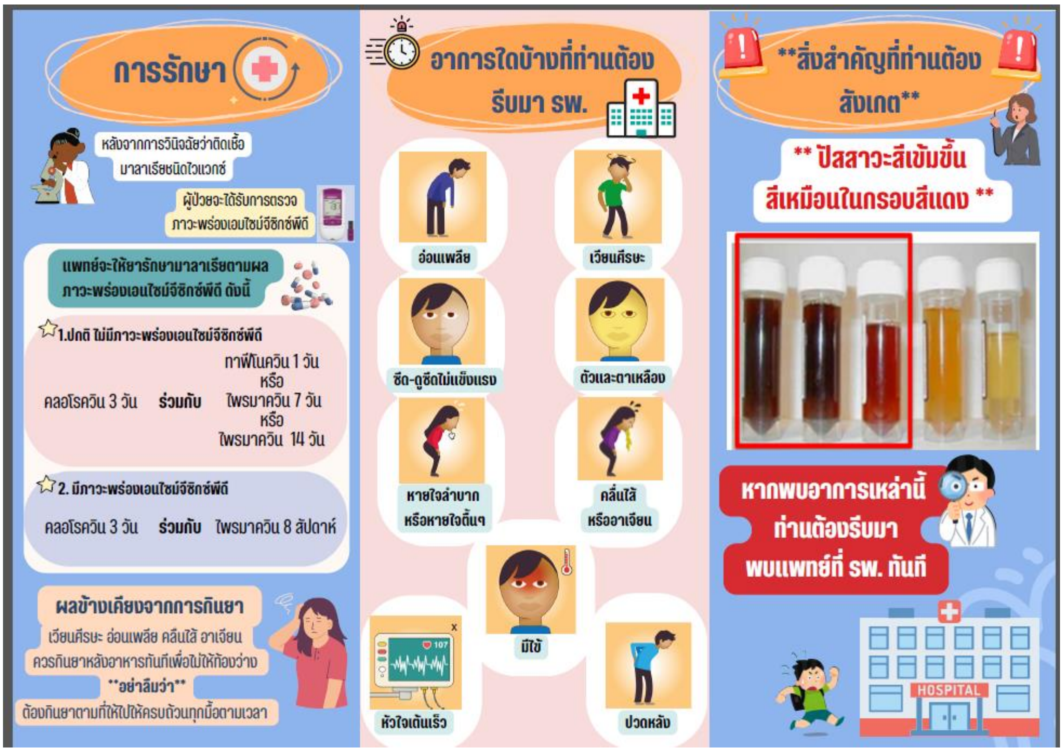

Figure S1. Quantitative G6PD testing results

A) G6PD status by treatment group; B) G6PD status by sex; C) distribution of G6PD enzyme activity by sex.

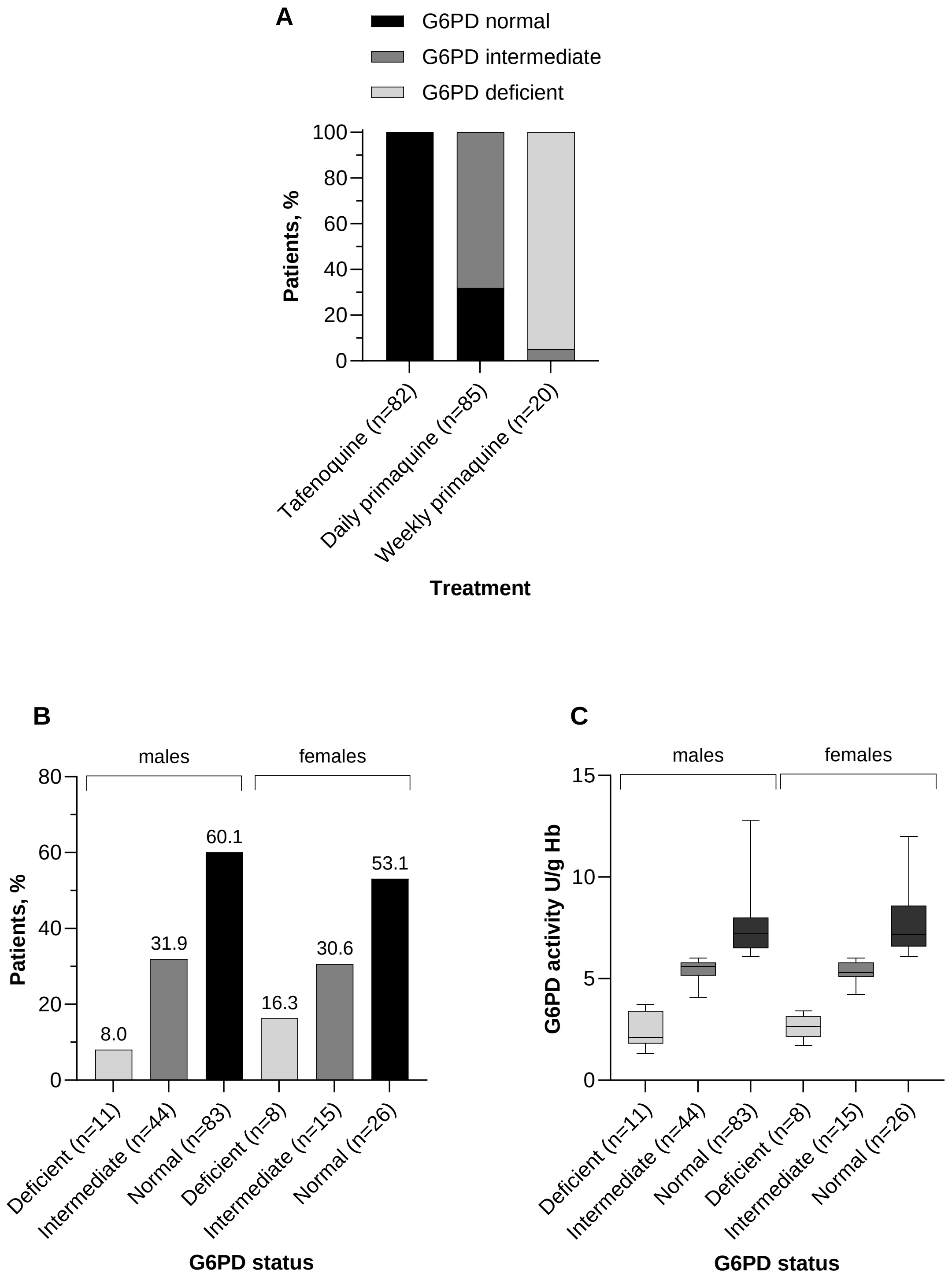

Table S1. Patients with suspected acute haemolytic anaemia at the day 5 follow-up visit (post-hoc analysis)

Treatment	Facility type	ID	Age	Gender	Baseline G6PD	Baseline haemoglobin	Day 5 symptoms	Day 14 symptoms	Drug-related AHA confirmed
Tafenoquine	Malaria clinic	417	31	Male	8.1	12.8	Dark urine, fatigue, dizziness, breathlessness, nausea, fever	None	No
	Hospital	701	38	Male	6.4	12.7	Pallor, fatigue	None	No
	Hospital[a]	426	37	Female	6.6	12.3	Fatigue, dizziness, back pain, jaundice, pallor, nausea	Fatigue, back pain	No
	Hospital	302	51	Female	8.7	11.1	Dark urine, fatigue, nausea	Pallor	No
	Hospital	431	26	Male	6.9	15.3	Fatigue, jaundice, pallor, fever	None	No
	Hospital	908	31	Male	7.6	14	Dark urine, back pain	None	No
Daily primaquine	Hospital	434	53	Male	5.5	13.1	Dark urine, fatigue, jaundice	None	No
	Hospital	415	51	Male	5.6	11.7	Dark urine, fatigue, dizziness, breathlessness, back pain, pallor, fever, nausea	None	No
	Hospital	420	27	Male	6.1	13.9	Dark urine, fatigue, back pain, nausea	None	No
	Hospital	418	52	Female	6.5	14.1	Dark urine, fatigue, nausea	Back pain	No
	Hospital	416	46	Female	4.2	11.9	Dark urine, fatigue, dizziness	Fatigue, back pain, fever	No
	Hospital	422	24	Male	5.5	14.5	Dark urine, fatigue, fever	None	No
	Hospital	303	25	Male	5.9	12.8	Jaundice, back pain	None	No
Weekly primaquine	Hospital	839	39	Male	2.7	14.8	Dark urine, fatigue, dizziness, nausea	Dizziness	No

AHA, acute haemolytic anaemia.

[a] Diagnosed with scrub typhus co-infection.

There were no patients with suspected acute haemolytic anaemia at the day 14 follow-up visit.

Table S2. Other adverse events reported at day 5 and day 14 follow-up visits

Adverse event	Day 5			Day 14		
	Tafenoquine (n=80)	Daily primaquine (n=81)	Weekly primaquine (n=18)	Tafenoquine (n=77)	Daily primaquine (n=77)	Weekly primaquine (n=17)
Headache	7	3	2	3	0	0
Anorexia	1	0	0	0	0	0
Cough/ sore throat	0	2	0	0	3	0
Rash	0	1	0	0	0	0
Tired	0	0	0	0	1	0
Diarrhoea	0	0	1	0	0	0

Figure S2. Adverse events of any nature reported by patients during day 5 and day 14 follow-up visits by facility level

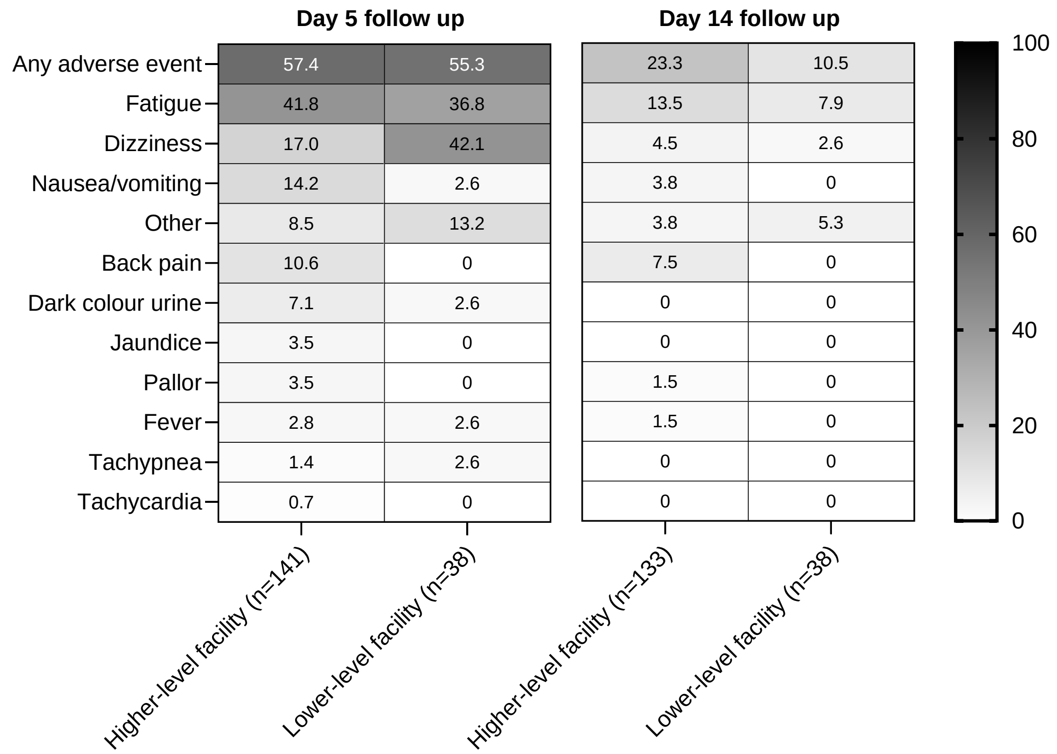

Figure S3. Adverse events of any nature reported by patients during day 5 and day 14 follow-up visits by sex

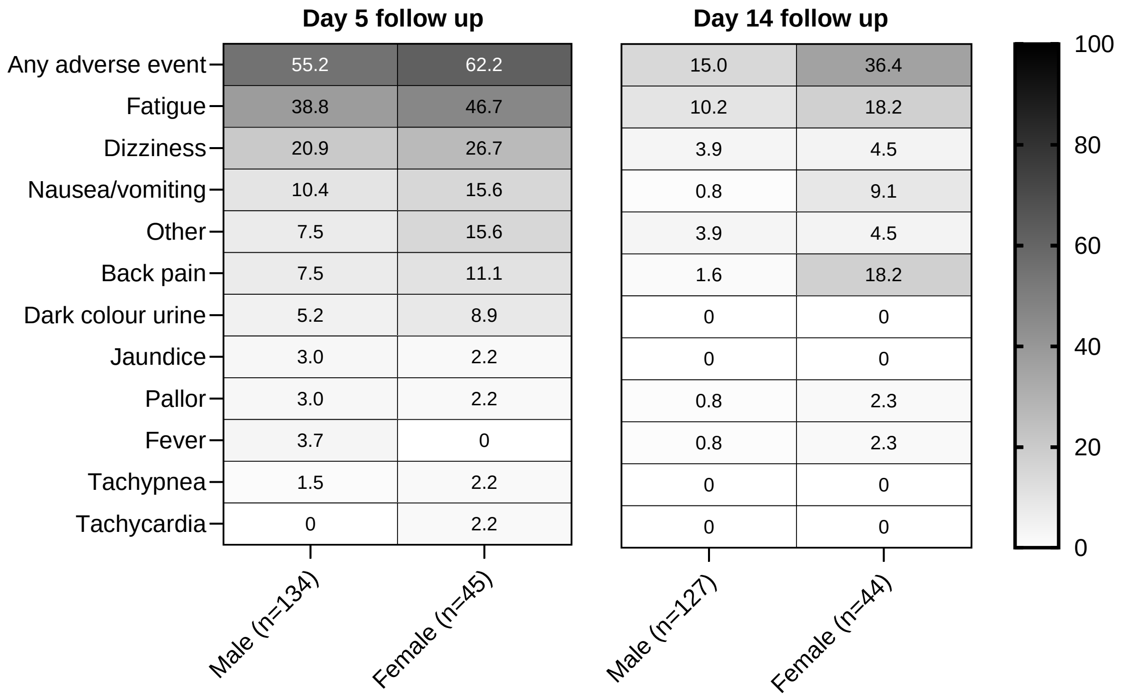

S1 Narratives. Safety narratives for patients with serious adverse events

Tafenoquine: Patient ID701

Serious adverse event for prolongation of hospitalisation in a male patient, 38 years, with malaria symptoms since 13 August 2022, diagnosed with *P. vivax* malaria on 19 August 2022 (D0) at Yala Vector Borne Diseases Centre and referred to Kabang hospital for confirmation of diagnosis and treatment. The patient asked to be admitted at Mayo hospital in hometown (Pattani province), where he was hospitalized the same day with symptoms of fever, chill, and weakness, as well as anaemia based on clinical laboratory reports. The patient was treated with tafenoquine and 3-day chloroquine on 19 August 2022 (D0). Considering the laboratory findings, the physician suspected Thalassemia-trait on 22 August 2022 and requested haemoglobin typing. The patient was discharged on 23 Aug 2022 (D4) with a plan for re-admission for blood transfusion when blood match was found at regional blood bank. Thalassemia-trait was confirmed by the presence of haemoglobin E on 25 August 2022 (D6). The patient was hospitalized again on 25 August 2022 (D6) as planned. Haemoglobin was 9.1 g/dL, haematocrit 27.4% and platelet count at 353,000/mcL. After re-admission, the patient received one unit of leukocyte-poor red cells (LPRC) and was discharged on 26 August 2022 (D7). The patient only complained about fatigue at the routine day 5 follow-up visit at Kabang hospital on 24 August 2022 (D5) and had no complaints by the day 14 visit on 2 September 2022 (D14). On that date, haemoglobin was 10.7 g/dL, haematocrit 32.6%, and platelets 423,000/mcL.

Case conclusion: This case of fever, chill, and weakness, as well as anaemia is reported as a serious adverse event because of the prolonged hospitalisation. The patient was diagnosed with Thalassemia-trait and was transfused. He was discharged after recovery. The serious adverse event was not considered related to antimalarial treatment by the physician.

**Laboratory exams (normal)**	**D0 (diagnosis) 19Aug22**	**D0 (hospitalised) 19Aug22**	**D1 20Aug22**	**D2 21Aug22**	**D3 22Aug22**	**D4 (discharge) 23Aug22**
G6PD activity (SD Biosensor)	6.4 U/g Hb					
RBC (3.9 to 5.7 million/mcL)	ND	ND	ND	ND	ND	ND
Hb (11.0 to 17.0 g/dL)	12.7	ND	9.2	8.4	8.5	8.3
Hct (36.0 to 50.0%)	ND	28.0	27.3	24.2	24.5	24.2
Platelet count (150 – 450/mcL)	ND	37	24	87	93	172
Total bilirubin (0.00 to 1.1 mg/dL)	ND	1.16	ND	ND	ND	ND
Direct bilirubin (0.0 – 0.25 mg/dL)	ND	0.71	ND	ND	ND	ND
BUN (7 – 18 mg/dl)	ND	26.4	26.4	16.7	12.1	ND
Creatinine (0.7 to 1.7mg/dL)	ND	1.16	1.4	1.04	1.03	ND
AST (0 to 40 UI/L)	ND	19	ND	ND	ND	ND
ALT (5 to 40 UI/L)	ND	15	ND	ND	ND	ND
Dengue rapid test IgG			Positive			

G6PD, glucose-6-phosphate dehydrogenase; RBC, red blood cells; Hb, haemoglobin; Hct, haematocrit; BUN, blood urea nitrogen; AST, aspartate aminotransferase; ALT, alanine aminotransferase.

Tafenoquine: Patient ID811

A serious adverse event in a male patient, 31 years old, with history of fever, severe headache and myalgia with a low platelet count. *P. vivax* malaria was diagnosed on 24 June 2022 (D0) in Mae Sariang hospital, presenting malaria symptoms since 22 June 2022. Treated with 3-day chloroquine, single-dose tafenoquine and ceftriaxone IV for prophylaxis of sepsis. He was hospitalised with fluctuating white blood cells count: 4,830/mcL on 23 June 2022 (D-1) to 15,900/mcL on 24 June 2022 (D0); and a decreased platelet count: 110,000/mcL on 23 June 2022 (D-1) to 92,000/mcL on 24 June 2022 (D0) to 81,000/mcL on 25 June 2022 (D1). Assessment for possible concomitant infections at the time of admission showed that the patient was negative for dengue and scrub typhus and had a Leptospirosis infection. The patient was treated with an additional antibiotic (doxycycline for 7 days). The physician concluded that symptoms, high white blood cell count and low platelet count on 24 June 2022 were possibly due to the Leptospirosis infection. After hospitalisation for 2 days, the patient's symptoms improved, and he was discharged from hospital on 25 June 2022 (D1). At the follow up visit on 27 June 2022 (D3), the patient had only mild headache, his complete blood count was within normal limits except for platelet count (126,000/mcL). The patient had no complaints of any signs of possible adverse events related to haemolysis on follow-up visits on day 5 or day 14.

Case conclusion: This case of fever, severe headache, and myalgia was reported as a serious adverse event because it was considered by the physician as a significant event in the initial description of the case. The patient had a leptospirosis infection without signs of acute haemolytic anaemia. He was discharged after recovery. The serious adverse event was not considered related to antimalarial treatment by the physician.

**Laboratory exams (normal)**	**D0 (diagnosis) 24Jun22**	**D0 (hospitalized) 24Jun22**	**D1 (discharged) 25Jun22**	**D3 (follow-up) 27Jun22**
G6PD activity (SD Biosensor)	6.6 U/g Hb	ND	ND	ND
WBC (4.0 – 10.0 1000/cumm)	ND	15.89	4.64	4.49
RBC (3.9 to 5.7 million/mcL)	ND	4.68	4.73	5.09
Hb (11.0 to 17.0 g/dL)	12.7	13.7	13.9	15.3
Hct (36.0 to 50.0%)	ND	42.4	43.5	45.8
Platelet count (150 – 450/mcL)	ND	92	81	126
Total bilirubin (0.00 to 1.1 mg/dL)	ND	0.52	ND	ND
Direct bilirubin (0.0 – 0.25 mg/dL)	ND	0.12	ND	ND
BUN (7 – 18 mg/dl)	ND	11.47	ND	ND
Creatinine (0.7 to 1.7mg/dL)	ND	1.08	ND	ND
AST (0 to 40 UI/L)	ND	ND	ND	ND
ALT (5 to 40 UI/L)	ND	ND	ND	ND
Leptospira-Ab		IgM Positive, IgG Negative		
Scrub typhus rapid test		Negative		
Dengue NS1 Antigen		Negative		

G6PD, glucose-6-phosphate dehydrogenase; WBC, white blood cell count; RBC, red blood cells; Hb, haemoglobin; Hct, haematocrit; BUN, blood urea nitrogen; AST, aspartate aminotransferase; ALT, alanine aminotransferase.

Tafenoquine: Patient ID417

A serious adverse event for hospitalisation in a male patient, 31 years old, diagnosed with *P. vivax* malaria at Mae Sariang malaria clinic on 4 July 2023 (D0), with G6PD activity of 8.1 U/g Hb and haemoglobin of 12.8 g/dL (SD Biosensor), treated with chloroquine and tafenoquine. The patient was hospitalised at Mae Sariang hospital on 5 Jul 2023 (D1), with fever, diarrhoea (10 times, no mucous-bloody stool), fatigue, vomiting after meal, dizziness, dark urine, oxygen saturation 95%, haemoglobin 16 g/dL, haematocrit 49.7%, white blood cell count 11,770/mcL, red blood cell count 5.67 x 106/mcL, and platelets 78,000/mcL. The patient was positive for scrub typhus rapid test (IgM positive, IgG negative), negative for dengue and Leptospira. Urinalysis: yellow, clear, albumin 3+, blood negative, blood urea nitrogen 18.67 mg/dL, creatinine 1.04 mg/dL, estimated glomerular filtration rate 95.22, sodium 127.8 mEq/L, and potassium 3.3 mEq/L. The diagnosis was confirmed for *P. vivax* malaria with scrub typhus co-infection. In addition, the patient suffered of hyponatraemia and hypokalaemia. Diarrhoeal symptoms in this patient could have been a side effect of tafenoquine or scrub typhus infection. However, the clinical diarrhoea improved rapidly after treatment with doxycycline (5 July 2023/10 times VS 6 July 2023/2 times), more in favour of a diarrhoea caused by the co-infection. The patient was discharged on 7 July 2023 (D3) after recovery.

Case conclusion: This case of fever, diarrhoea, dizziness is reported as a serious adverse event because of the hospitalisation. The patient had a scrub typhus co-infection without signs of acute haemolytic anaemia. He was discharged after recovery. The serious adverse event was not considered related to antimalarial treatment by the physician.

Daily primaquine: Patient ID810

A serious adverse event for prolongation of hospitalisation in a 68-year-old female patient diagnosed with *P. vivax* malaria on 16 June 2022 (D0) in Mae Sariang hospital, presenting malaria symptoms since 15 June 2022. Patient was referred from a malaria clinic following a malaria smear positive for *P. vivax*, G6PD activity was 5.3 U/g Hb and haemoglobin of 12.9 g/dL (SD Biosensor). On admission, haemoglobin was 10.8 g/dL, red blood cells 3.85, haematocrit at 33.3%, and platelet count 155,000/mcL. Treated with chloroquine for 3-days and primaquine for 14-days from 16 June 2022 (D0) with double-dose primaquine given by mistake on D5 for 1 day. Prolongation of hospitalisation was requested on 20 June 2022 (D4) by the physician due to 'severe malaria' with severe thrombocytopaenia.

Transfusion with four units of (leukocyte depleted) pooled platelet concentrate on 22 June 2022 (D6) and six units on 25 June 2022 (D9). The patient was discharged on 28 June 2022 (D12). The patient was seen at the first follow-up visit on 04 July 2022 and the physician started a treatment with prednisolone (1 mg/kg/day) for idiopathic thrombocytopenic purpura (Coomb's test repeated on 27 June 2022 result not shown, previous direct Coomb's was 2+). Prednisolone was stopped on 12 September 2022.

Case conclusion: This case of 'severe malaria' with severe thrombocytopenia is reported as a serious adverse event because of the prolonged hospitalisation. The patient was transfused then treated for an idiopathic thrombocytopenic purpura. The serious adverse event was not considered related to antimalarial treatment by the physician.

(see over for investigations)

Date	D0 16Jun22	D1 17Jun22	D2 18Jun22	D3 19Jun22	D4 (hospitalised) 20Jun2022	D4 20Jun22 repeated	D4 20Jun22 repeated	D6 22Jun22 before transfusion
Platelets/mcL	155K	111K	69K	28K	**8K**	3K	2K	7K
Hb g/dL	ND	ND	NDD	NA	**10.8**	10.1	9.9	ND
	**D7 23Jun22**	**D8 24Jun22**	**D9 25Jun22 before transfusion**	**D9 25Jun22 after transfusion**	**D10 26Jun22**	**D11 27Jun22**	**D12 discharged 28Jun22**	**D19 04Jul22**
Platelets/mcL	14K	12K	15K	29K	25K	20K	33K	49K
	**D8 24Jun22**	**D9 25Jun22 before transfusion**	**D9 25Jun22 after transfusion**	**D10 26Jun22**	**D11 27Jun22**	**D12 discharged 28Jun22**	**D19 04Jul22**	**D25 10Jul22**
Platelets/mcL	12K	15K	29K	25K	20K	33K	49K	171K
	**D33 18Jul22**	**D46 31Jul22**	**D47 01Aug22**	**26Sep22**	**27Oct22**			
Platelets/mcL	169K	53K	59K	207K	191K			

Hb, haemoglobin.
